# p53: A player in the tumor microenvironment

**DOI:** 10.32604/or.2025.057317

**Published:** 2025-03-19

**Authors:** SHUANG ZHAO, HONGYONG WEN, BAIQI WANG, QINGLIN XIONG, LANXIN LI, AILAN CHENG

**Affiliations:** 1 Hunan Engineering Research Center for Early Diagnosis and Treatment of Liver Cancer, Cancer Research Institute, Hengyang Medical School, University of South China, Hengyang, 421001, China; 2 The Second Affiliated Hospital, Hengyang Medical School, University of South China, Hengyang, 421001, China

**Keywords:** Tumor microenvironment (TME), p53, Wild type p53, Mutant p53, Cancer therapy

## Abstract

Approximately half of all cancers have p53 inactivating mutations, in addition to which most malignancies inactivate the p53 pathway by increasing p53 inhibitors, decreasing p53 activators, or inactivating p53 downstream targets. A growing number of researches have demonstrated that p53 can influence tumor progression through the tumor microenvironment (TME). TME is involved in the process of tumor development and metastasis and affects the clinical prognosis of patients. p53 participates in host immunity and engages in the immune landscape of the TME, but the specific mechanisms remain to be investigated. This review briefly explores the interactions between different states of p53 and TME components and their mechanisms, as well as their effects on tumor progression. To understand the progress of drug development and clinical studies related to p53 and tumor microenvironment.

## Highlights:

1. Interactions between wild-type and mutant p53 and components within the tumor microenvironment and their impact on tumor progression.
2. A description of the progress of drug development and clinical studies related to p53 and the tumor microenvironment, providing a basis for further research.

## Abbreviations

Bac^Mel^: Photothermal melanin
BAI1: Brain angiogenesis inhibitor 1
bFGF: Basic fibroblast growth factor
CAFs: Cancer-associated fibroblasts
CAR: Chimeric antigen receptor
CCL2: Chemokine (C-C motif) ligand 2
CCR2: CC chemokine receptor 2
COX-2: Cyclooxygenase-2
CSF1: Colony-stimulating factor 1
CTD: C-terminal domain
CTL: Cytotoxic T-lymphocyte
CTLA-4: Cytotoxic T-lymphocyte antigen 4
CXCL1, CXCL2 and CXCL3: Melanoma growth stimulatory activities
CXCL5: Epithelial neutrophil activating protein
CXCL8: Interleukin-8
CXCL17: Chemokine (C-X-C motif) ligand 17
CXCR3: CXC chemokine receptor 3
cGAS: Cyclic GMP-AMP synthase
DAB2IP: Disabled homolog-2 interacting protein
DBD: DNA binding domain
DMBA: 7,12-dimethylbenz[a]anthracene
dMMR: Deficient mismatch repair
ECM: Extracellular matrix
ELR: Glu-Leu-Arg
EMT: Epithelial-to-mesenchymal transition
FGF10: Fibroblast growth factor 10
Foxp3: Transcription factor forkhead box protein 3
GOF: Gain-of-function
HIF-1**α**: Hypoxia-inducible factor-1 alpha
HLA-A: Human leukocyte antigen-a
H2: Bispecific antibody
ICIs: Immune checkpoint inhibitors
ID4: Inhibitor of DNA binding 4
IFN-Is: Type I interferons
IL6: Interleukin 6
IRF3: Interferon regulatory factor 3
KPC: Kras LSL-G12D/þ; Trp53 LSL-R172H/þ; Pdx1-Cre
LAG-3: Lymphocyte activation gene 3
MAPK: Mitogen-activated protein kinases
MCP-1: Monocyte chemoattractant protein-1
mCRC: Metastatic colorectal cancer
MDM2: Murine double minute 2
MSCs: Mesenchymal stem cells
MSI-H: High microsatellite instability
NF-κB: Nuclear factor kappa-B
NK: Natural killer
NSCLC: Non-small cell lung cancer
PCs: Pancreatic cancer cells
PD-1: Anti-programmed cell death protein 1
PD-L1: Programmed cell death ligand 1
PGE2: Prostaglandin E2
PKC: Protein kinase C
PMNs: Polymorphonuclear cells
PRD: Proline-rich domain
PTGS2: Prostaglandin Endoperoxide Synthase 2
SASP: Senescence-associated secretory phenotype
SIRPα: Signal regulatory protein alpha
SP1: Specificity protein 1
STAT3: Signal transducer and activator of transcription 3
STAT5: Signal transducer and activator of transcription 5
STING: Stimulator of interferon genes
TAD: Transactivation domains
TAMs: Tumor-associated macrophages
TANs: Tumor-associated neutrophils
TBK1: TANK-binding kinase 1
TIGIT: T-cell immunoglobulin and the ITIM structural domain
TGF-β: Transforming growth factor-beta
TNBC: Triple-negative breast cancer
Th: CD4^+^T helper
TNF-α: Tumor necrosis factor-alpha
Tregs: Regulatory T cells
TREX1: Three-prime repair exonuclease
TRIM24: Tripartite motif-containing 24
TME: Tumor microenvironment
VEGF: Vascular endothelial growth factor
VPF: Vascular permeability factor
OD: Oligomerization domain
OS: Overall survival
ROS: Reactive oxygen species
RFS: Relapse-free survival
YTHDF2: YTH domain family 2
ZMC-1: Zinc metallochaperone-1
LPS: Lipopolysaccharide
α-SMA: Alpha-smooth muscle actin

## Introduction

p53 consists of 393 amino acids, which has a molecular weight of approximately 53 KD. p53 has seven major functional domains. The central DNA binding domain (DBD) and the regulatory C-terminal domain (CTD) mediate the binding of p53 to DNA. The oligomerization domain (OD) is required for the formation of p53 tetramers, which is a prerequisite for p53 activity as a transcriptional activator. In addition, p53 has two transactivation domains (TAD1 and TAD2) located at the n-terminus and a proline-rich domain (PRD) [1]. The transcription factor p53 plays an important role in regulating cellular responses such as cell cycle control, differentiation, apoptosis, DNA repair and proliferation, as well as mediating the tumor microenvironment (TME) [2].

The tumor microenvironment is a complex and evolving process. The tumor microenvironment includes innate immune cells, adaptive immune cells, stromal cells, fibroblasts and endothelial cells [3]. Tumors can modify their surroundings by secreting a variety of cytokines, chemokines, and other factors [3]. The theory of immune surveillance suggests that the immune system can control tumor growth. In the tumor microenvironment, those involved in immune surveillance are CD4^+^ T helper (Th) cells, CD8^+^ cells, natural killer (NK) cells, and some neutrophils [4]. However, other subtypes of the immune system have a role in supporting tumor progression [4]. For example, regulatory T cells (Tregs) promote tumor growth by suppressing CD4^+^ and CD8^+^ T cell activity [5]. Macrophages and other polymorphonuclear cells (PMNs) can promote angiogenesis, metastasis, and immunosuppression by regulating inhibitory cytokines and surface ligands [6]. Both the acquisition and maintenance of cancer characteristics are associated with TME [7]. In conclusion, TME is a crucial material basis for tumor occurrence, development, and metastasis.

In recent years, how p53 affects the mechanism of tumor growth inhibition has been the focus of research [8]. In the tumor microenvironment, p53 alters the immune landscape and thus exerts anti-tumor effects [9]. Different states of p53, such as wild-type p53, mutant p53, p53 inactivation and p53 deletion, play various roles in the tumor microenvironment. Among them, mutant p53 is more invasive and metastatic compared to wild-type p53 and p53 deletion [9]. Due to the increasing importance of TME in cancer biology, the direction of cancer research and treatment has shifted from cancer-centered to TME-centered [10]. Therefore, it is necessary to understand the mechanism of p53-mediated TME. This review will focus on the interactions between p53 and TME components from the perspective of different states of p53 and further explore the progress of p53 in clinical and pharmaceutical research.

## The Role of p53 to Regulate Tumor Microenvironment

(1) Chronic inflammation-related mechanism

Inflammation is an immune defense response of the body to stimulation. However, tumor development is closely linked to an excessive immune response. Chronic inflammation strikes the immune balance and provides certain external conditions for tumor development, progression and metastasis [11]. A growing body of research demonstrates that p53 regulates crosstalk in the tumor immune system [12]. p53 plays a role in multiple inflammatory pathways.

p53 interacts with a variety of inflammatory factors. Studies have reported that p53 has extensive interactions with inflammatory factors such as reactive oxygen species (ROS), nuclear factor kappa-B (NF-κB) and signal transducer and activator of transcription 3 (STAT3) [13–15]. It has been reported that p53 can inhibit the NF-κB pathway by either directly inhibiting the promoter activity of p65 or indirectly inhibiting the activity of the I-κB α kinase IKKα [16]. Wild-type p53 antagonized NF-κB signaling inhibited the expression of cytokines and chemokines, and reduced the accumulation of ROS, thereby suppressing the inflammatory response in prostate cancer cells [17]. In addition, a recent study by Ghosh et al. demonstrated that WT p53 in tumor cells recruits the ubiquitin ligase tripartite motif-containing 24 (TRIM24) to promote the Three-prime repair exonuclease (TREX1) degradation, leading to cytoplasmic DNA accumulation, activation of the cytoplasmic DNA-sensing cyclic GMP-AMP synthase/stimulator of interferon genes (cGAS/STING) pathway, induction of type I interferons (IFN-Is), and tumor suppressor effects [18]. IFN-Is can also synergize with p53 to exert tumor-suppressive and stress-inducing effects [19]. All of the above studies suggest that p53 can influence the inflammatory response to tumor progression by interacting with inflammatory factors.

Most TP53 mutations fall into two broad categories. The first group of “DNA-contact” mutations consists of mutations in residues that are directly involved in DNA binding, such as R248Q and R273H. The second group of “conformational” mutations consists of mutations that cause local (e.g., R249S and G245S) or global (e.g., R175H and R282W) conformational aberrations [20]. In addition to inactivating the tumor suppressor function of p53, some of these mutations may confer functionally acquired properties on the mutant p53, including induced tumorigenic potential [21]. Notably, mutant p53 is common in inflammation-associated cancers, such as ovarian, lung, and colon cancers [22–24]. cGAS-STING-TANK-binding kinase 1-interferon regulatory factor 3 (TBK1-IRF3) signaling pathway leads to immune cell-mediated tumor suppression by recognizing DNA released into the cytoplasm during chromosomal instability thereby regulating gene expression and inducing apoptosis. Mutant p53 binds TBK1 and disrupts downstream signaling from cGAS/STING to TBK1, thereby preventing phosphorylation of its substrates and inhibiting IRF3 activation *in vivo* to promote immune evasion [25]. In addition, active STING signaling generates type I IFN, which is essential for initiating anti-cancer immune responses [25]. IFN-I signaling activates cytotoxic T lymphocytes (CTL) by upregulating the expression of MHC-I, MHC-II, and co-stimulatory molecules (e.g., CD40, CD80, and CD86), releasing its antigen-presenting capacity [26]. In addition, inflammation induces mutation and inactivation of p53, which in turn exacerbates the chronic inflammatory response in tumor tissues and organs, thereby promoting tumor development and metastasis [12].

The main cause of p53 inactivation is mutations in the p53 DBD, which prevents the protein from binding to target DNA [27]. p53 inactivation triggers overactivation of the NF-κB pathway in cells, which induces an increased secretion of inflammatory cytokines and chemokines, ultimately exacerbating chronic inflammation and tumorigenesis [28].

The p53 deletion is a result of a genetic aberration. Deletion of p53 in cancer cells increases ROS levels, activates STAT3 signaling, and regulates the expression of inflammatory cytokines including monocyte chemoattractant protein-1 (MCP-1), interleukin 6 (IL6), and colony-stimulating factor 1 (CSF1) [17]. Mutant p53 interacts with disabled homolog-2 interacting protein (DAB2IP) in the tumor necrosis factor-alpha (TNF-α) signaling pathway to regulate tumor cell responses to inflammatory signals and promote inflammation-associated tumorigenesis [17].

(2) Extracellular matrix (ECM)-related mechanism

ECM consists of collagens, proteoglycans/glycosaminoglycans, elastin, fibronectin, laminins, and several other glycoproteins [29]. ECM plays a crucial role in cancer cell attachment, growth, and even sensitivity to chemotherapy and cellular drug therapy. During tumor evolution, cancer cells and cancer-associated fibroblasts (CAFs) act together to alter the biochemical, physical, and biomechanical properties of the ECM occurring through increased matrix secretion and remodeling [7].

Mutant p53 alters the structure of the ECM through different mechanisms. Indeed, missense mutant p53, which together with hypoxia-inducible factor-1 alpha (HIF-1α) is located at its target chromatin locus, recruits SWI/SNF chromatin remodelers and leads to the hyperactivation of a specific subpopulation of HIF-regulated genes encoding ECM components [4]. The mut-p53/HIF-1α/miR-30d axis enhances the release of soluble factors and the deposition and remodeling of the ECM, affecting mechanosignaling and stromal cell activation in the tumor microenvironment, thereby promoting tumor growth and metastatic colonization [30]. Dysregulation of ECM remodeling, which is locally characterized by excessive ECM deposition and increased stiffness, has been shown to have an important effect on cell fate under various fibrotic conditions [31]. As tumor cells proliferate, the surrounding ECM undergoes significant structural changes in the dynamic interactions between the microenvironment and resident cells. These changes, including increased secretion of fibronectin and collagen I, III, and IV. Increased matrix protein deposition promotes tumor progression by interfering with cell-cell adhesion, cell polarity and ultimately amplifying growth factor signaling [31]. In contrast, in non-small cell lung cancer, mutant p53 interacts with HIF-1α, leading to an increase in collagen VIIa1 and laminin-γ2 in ECM, which worsens prognosis [32]. It has been shown that in pancreatic cancer, gain-of-function (GOF) mutant p53 induce CAFs to secrete heparan sulfate proteoglycan 2 (HSPG2, or perlecan), an ECM proteoglycan. This activity is required for invasive metastasis of functionally acquired mutant p53 and p53-deficient cancer cells [33]. Taken together, p53 mutations promotes tumor invasion and metastasis by remodeling the ECM.

(3) Tumor-infiltrating T cells-mediated mechanism

Tumor cells can evade immune surveillance by reducing T cell infiltration through epigenomic reprogramming or by constructing a suppressive tumor microenvironment [34]. Among tumor-infiltrating T cells, CD8^+^ T cells play a central role in immunotherapy-induced tumor immunity therefore tumor immunity is one of the determinants of outcome and prognosis in cancer patients [35]. However, Tregs have the ability to inhibit CD4^+^ and CD8^+^ T cell activity and thus promote tumor growth [4]. Tumor-infiltrating T cells typically enter a dysfunctional state known as T cell exhaustion [36]. Depleted T cells are highly expressive of inhibitory surface molecules known as immune checkpoints, which prevent T cell activation. These include anti-cytotoxic T lymphocyte-associated antigen-4 (CTLA-4), anti-programmed cell death protein 1/programmed cell death ligand 1 (PD-1/PD-L1), lymphocyte activation gene 3 (LAG-3), T-cell immunoglobulin and the ITIM structural domain (TIGIT) [37]. However, tumor cells can avoid recognition by the immune system through immune evasion mechanisms such as up-regulation of immune checkpoints to suppress T-cell effects [38].

Several studies have shown that wild-type p53, as a negative regulator of autoimmunity, affects the immune response in multiple ways. Under inflammation-promoting conditions, p53 inhibits the STAT3-Th17 axis but promotes the development of the signal transducer and activator of transcription 5 (STAT5)-Treg axis to support Tregs, thereby suppressing the autoimmune response [39,40]. For example, in human colon and breast cancer cells, p53 upregulates transcription factor forkhead box protein 3 (Foxp3), a major regulator of Tregs, and suppresses autoimmunity [41,42]. This suggests that p53 expressed in T cells, p53 can suppress autoimmune responses by supporting Tregs and inhibiting the differentiation of Th17 cells [41,43]. Mouse double minute 2 (MDM2) is a key negative regulator of the tumor suppressor p53 protein, inhibiting p53 protein activity and decreasing p53 concentration through a p53-MDM2 feedback loop [44]. It has been shown that the MDM2-c-Cbl-STAT5 axis plays a critical role in CD8^+^ T cell function. p53-MDM2 pathway promotes CD8^+^ T cell-mediated anti-tumor immunity by competitively binding to STAT5, thereby increasing the stability of STAT5 [45].

Patients with p53 mutations exhibit a non-T-cell infiltrating phenotype in which cytotoxic T cells, helper T cells and NK cells are reduced. In contrast, highly immunosuppressed Tregs and M2 macrophages were expanded [46]. Mutated p53 induces Treg differentiation and a shift towards inflammation. Recent studies have shown that inactivation of p53 in tumor cells suppresses effector CD4^+^ and CD8^+^ T cells, thereby enhancing the suppressive function of Tregs and myeloid suppressor cells [12].

Inactivation of p53 in immune cells enhances inflammation-induced tumorigenesis in a number of ways, e.g., by enhancing the production of chemokines and inflammatory cytokines, promoting the differentiation and function of Th17 cells, and inhibiting the differentiation of Tregs, thereby altering the balance between inflammation and inflammatory tolerance [39,41].

Studies using kras-driven pancreatic tumor-derived cancer cells as a model of p53 deficiency have shown that p53 deficiency improves immune tolerance by increasing myeloid cells and Tregs [40]. p53 deficiency induces recruitment of suppressive bone marrow CD11b^+^ cells and increases CXC chemokine receptor 3 (CXCR3)/CC chemokine receptor 2 (CCR2)-associated chemokines and macrophage colony-stimulating factor, decreasing CD4^+^ T helper cell 1 and CD8^+^ T cell responses *in vivo* [40,46]. Tregs are reported to be significantly increased in p53-deficient mice and are associated with accelerated tumor growth [47]. In addition, p53-deficient prostate cancer cells up-regulate the secretion of CXC chemokine receptor 17 (CXCL17), which is also associated with an increase in immunosuppressive Treg cells [40]. However, there are relatively few *in vivo* studies examining how the p53 status of cancer cells affects the immune response, and the p53-Tregs The relationship between p53 and Tregs is still controversial in cancer research.

(4) CAFs-mediated mechanism

CAFs are most abundant in stromal cells present in the TME [48]. Indeed, CAFs play a pivotal role in tumorigenesis, metastasis, immune escape, angiogenesis, and drug resistance by participating in intercellular contacts, secretion of a large number of regulatory molecules and extracellular vesicles, as well as synthesis and remodeling of ECM [7], such as pancreatic ductal adenocarcinoma [49], lung adenocarcinoma [50], among others [51]. A growing body of literature suggests that inhibition of p53 in CAFs leads to immune escape and perpetuates tumorigenesis [4,52,53]. We will elaborate on the following aspects.

Wild-type p53 inhibits tumor cell proliferation invasion and migration by affecting CAFs. Arandkar et al. reported that tumor cells could inhibit the induction of p53 in CAFs, which is required for tumor transformation [54]. Another study demonstrated that p53-mediated cellular senescence and senescence-associated secretory phenotype (SASP) of CAFs were associated with the proliferative potential of pancreatic cancer cells (PCs) and that treatment of CAFs with p53 inhibitors significantly inhibited the proliferation of PC cells [55]. In addition, Neta Moskovits et al. showed that wild-type p53 protein inhibits the expression and secretion of SDF-1 in stromal fibroblasts, which in turn inhibits the invasion and migration of its cognate receptor CXCR4 in tumor cells [12,56].

Loss-of-function and mutations in p53 may be important factors in the transformation of fibroblasts into CAFs. In Ma et al., transfection of fibroblasts with extracellular vesicles containing mutated p53 promoted the transformation of fibroblasts to CAFs and tumor growth [57]. There are other ways in which p53 interacts with CAFs. p53 mutations increase collagen contraction and upregulate CAFs-related markers, including CXCL12, fibroblast growth factor 10 (FGF10) and alpha-smooth muscle actin (α-SMA), by targeting and activating STAT3 [58]. This study suggests that p53 mutations in CAFs may alter the expression of CAFs secretagogues and exert the pro-cancer effects of CAFs. Yoshii et al. showed that mutations in the p53 gene in colon cancer cells inhibit p53 activity in fibroblasts and promote cell proliferation and its tumor support function [59]. Furthermore, it has been found that mutant p53 in CAF can apply selective pressure on neighboring epithelial cells for transformation [60]. Ju et al. found that the *in vivo* introduction of p53 mutants into HCT116 cells significantly enhanced lung metastasis [61]. The above studies suggest that p53 mutations not only promote the transformation of fibroblasts into CAFs, but also render CAFs pro-carcinogenic.

Inactivation of p53 affects CAFs. Cytokines and chemokines such as these factors CXCL1, CXCL12, IL-1β, and vascular endothelial growth factor (VEGF) enhance the tumor- and inflammation-promoting effects of p53 inactivation in CAFs [4]. p53 inactivation increases PD-L1 surface expression, which suppresses T cell function [4].

Some studies have reported that p53 deletion in CAF, hepatic stellate cells, or mesenchymal stem cells (MSCs) promotes the growth of cancer cells, including prostate cancer and liver cancers, etc. [4]. p53 mutation in CAFs promotes tumor cell migration and invasion [54].

(5) Tumor-associated macrophages-mediated mechanism

In TME, as a source of immunosuppressive cytokines and pro-tumor growth factors, tumor-associated macrophages (TAMs) are infiltrated and activated to express CSF1 and chemokine (C-C motif) ligand 2 (CCL2), thereby promoting tumor cell infiltration and metastasis [4,19,40,52]. Macrophages are classified into M1-like and M2-like phenotypes. Among them, the M1 type has a tumor suppressor role, in contrast to the M2-like phenotype, where macrophages have an immunosuppressive function that favors tumor growth, invasion, metastasis, and drug resistance. With changes in the environment, macrophages can switch between M1 and M2 phenotypes [17].

As the first transcription factor reported to have the ability to inhibit M2-type macrophage polarization, p53 has a dual role in regulating macrophage polarization. p53 is involved in the regulation of macrophage polarization and influences tumor development [62]. On the one hand, p53 inhibits NF-κB and State1 signaling and reduces pro-inflammatory genes in M1-type TAMs [63]. p53 can drive M1 macrophage polarization through induction of SASP [60]. NF-κB is involved in and plays a key role in the polarization of p53 by inducing SASP M1-type macrophages. Therefore, it can be said that p53 cooperates with NF-κB together to exert anti-tumor effects in TAMs [60]. On the other hand, p53 interacts with c-Myc to regulate M2-type macrophage polarization [63]. The mechanism by which p53 regulates M2-type macrophages has not been fully elucidated. It has been shown that both c-Myc and p53 are major hubs in the human M2 macrophage network, and that c-Myc acts downstream of p53 and reciprocally controls an overlapping subset of m2-specific genes with p53, and that c-Myc plays a key role in the M2 macrophage response to p53 activation [63]. It has been shown that c-Myc negatively regulates the promoter of BRD7, a transcriptional cofactor of p53 [64]. p53 downregulates M2 macrophage polarization through the p53/MDM2/c-Myc axis [63]. p21 may be one of the targets of p53 regulation of M2-type macrophages. p21, as a downstream of p53, is upregulated during M2-type macrophage polarization. However, deletion of p21 did not alter the role of nutlin-3 on macrophage polarization [63]. In addition, YTH structural domain family 2 (YTHDF2) was found to promote M2 polarization by destabilizing p53 mRNA [62,65]. In addition to regulating macrophage polarization, p53 can interact with macrophages in other ways. For example, p53 in macrophages leads to anti-inflammatory responses by inhibiting STAT1 [40]. In addition, p53 was found to regulate IL-4-stimulated M2 polarization in animal peritoneal macrophages in mouse experiments [63]. p53 can influence the innate immune system and suppress tumorigenesis by secreting factors that regulate macrophage function [66]. CD204, a class a scavenger receptor, is highly expressed in M2-polarized macrophages and CD204 (+) TAM and is associated with tumor progression in several cancers, including lung, pancreatic, ovarian, and gliomas [67]. Some studies have reported that p53 expression is associated with CD204+ TAMs and tumor vascular density in colorectal cancer, however, the exact mechanism needs to be further investigated [52,67].

However, mutant p53 can induce macrophages to enter the M2 state through multiple pathways. It has been shown that hematopoietic stem cells deficient in p53 induced macrophage differentiation toward the tumor-promoted M2 state [60]. Further studies found that tumor cells carrying p53 R273H or p53 R249S mutants induced macrophages to enter the M2 state, whereas tumor cells carrying other mutants, such as p53 R175H, did not undergo this phenotypic shift in macrophages [68]. M2 gene expression was significantly increased in M2-polarized macrophages from p53-mutant mice and p53-deficient [69]. Mutant p53 also induces macrophage conversion to M2-type via exosomes. Studies have reported that in enteritis occurring in colon cancer, p53 mutant colon cancer cells produce mir-1246-enriched exosomes. These exosomes can be taken up by neighboring macrophages, which conversely triggers the reprogramming of macrophages to the M2 type, and finally leads to immunosuppression promoting the induction and metastasis of colorectal cancer epithelial-to-mesenchymal transition (EMT) through transforming growth factor-beta (TGF-β) signaling [12,19,70]. In addition to inducing macrophages to enter the M2 state, p53 deletion in macrophages or mast cells has been shown to increase the production of pro-inflammatory cytokines [43]. Notably, the MAPK and NF-κB signaling pathways are key pathways in inflammation and M1 macrophage polarization [71]. Lipopolysaccharide (LPS) stimulation in p53-deficient mice promotes the production of pro-inflammatory cytokines by macrophages through modulation of NF-κB activity, reported in a study [72].

In addition, p53 deletion plays a role with in TAM. It has been shown that the response of TAM to p53 deletion is also increased in ovarian, lung, pancreatic and 7,12-dimethylbenz[a]anthracene (DMBA)-induced skin cancers [4]. p53 deletion increased expression of several M2 genes, increased arginase activity, and enhanced proliferation of M2 macrophages [63]. These tumorigenic, fibroblast stimulation of exosomes released by p53-deficient colorectal cancer cells can be explained by up-regulation of multiple exosomal mirna, including miR-1249-5p, miR-6737-5p, and miR-6819-5p, which all target TP53 mRNAs [73].

(6) Tumor-associated neutrophils (TANs)-mediated mechanism

TANs promote tumor progression, invasion, and angiogenesis. In response to cytokine stimulation, neutrophils have the ability to differentiate into either an antitumor-active N1 TAN or a tumor-promoting active N2 TAN [74,75]. N1 TANs exert antitumor activity through cytotoxicity. N2 TANs stimulate immunosuppression, tumor growth, angiogenesis and metastasis through DNA destabilization or release of cytokines and chemokines [74]. The immunological hallmark of N2 TAN is the upregulation of chemokines [74]. Neutrophil recruitment is mainly mediated by chemokines with glutamate-leucine-arginine motifs (ELR^+^ chemokines), mainly melanoma growth-stimulating activity (CXCL1, CXCL2), epithelial neutrophil activating protein (CXCL5), interleukin-8 (CXCL8) [74,76]. CXC can be subdivided into ELR+ CXC and ELR-CXC chemokines based on the presence or absence of the Glu-Leu-Arg (ELR) tripeptide motif at the end of NH2 [74,77]. ELR+ chemokines are characterized by their ability to specifically recruit neutrophil polymorphonuclear leukocytes (PMN) into inflamed tissue [77]. Limited by the technical constraints of obtaining TANs from human tumor tissues, so far, the polarization of human TANs and their dual effects on tumor cell growth and metastasis remain controversial [74].

Mutant p53 is associated with tumor-associated neutrophils. It has been reported that the gain-of-function mutant Trp53 R172H causes tumor-associated neutrophil infiltration [75]. Mutations in the p53 gene may mediate an increase in ELR^+^ chemokines, which recruit neutrophils [75]. Several studies have suggested that the p53 R172H mutation may increase CXC chemokine levels through NF-κB. Studies on the Kras^LSL-G12D/+^; Trp53^LSL-R172H/+^; Pdx1-Cre (KPC) mouse model showed that the mutant Trp53 R172H effectively increased CXCL5 levels through the activation of NF-κB [75]. In cancers including breast, lung, and melanoma, increased CXCL5 transcription depends on the acquisition of a functional mutant p53 [78]. Furthermore, analysis of pancreatic cancer showed that expression of genes related to the NF-κB inflammatory pathway was significantly enriched in tumors with high CXCR2 ligand expression [79]. All of these studies suggest that mutant p53 induces an increase in chemokines through activation of NF-κB, which promotes the recruitment of tumor neutrophils and thus suppresses T cell responses [4].

p53 deletion also affects TANs. It has been shown that in a mouse model of breast cancer, p53 gene deletion increases neutrophil levels through uncontrolled WNT signaling, leading to metastasis [80].

(7) Angiogenesis-related mechanism

Angiogenesis is one of the most fundamental processes in tumor growth, which promotes tumor invasion and metastasis to peripheral and distant tissues. p53 negatively regulates the angiogenic process through a variety of mechanisms. Under hypoxic conditions, specificity protein 1 (SP1) binds to the vascular permeability factor (VPF)/VEGF promoter and promotes angiogenesis in breast cancer cells [81].

p53 has an inhibitory effect on angiogenesis. It has been reported that wild-type p53 binds to SP1 protein and prevents it from interacting with the VPF/VEGF promoter, thereby inhibiting angiogenesis [81]. Reportedly, p53 participates in the inhibition of angiogenesis through transcriptional regulation of brain angiogenesis inhibitor 1 (BAI1) [82]. Studies have shown that wild-type p53 inhibits angiogenesis through proteasomal degradation of HIF-1α [81]. According to different studies, wild-type p53 inhibits tumor angiogenesis by decreasing the levels of basic fibroblast growth factor (bFGF) and cyclooxygenase-2 (COX-2) in addition to VEGF [81].

However, p53 dysfunction can induce angiogenesis [81,83]. Mutant p53 participates in the maintenance of angiogenesis. It has been reported that mutp53 R175H and R273H bind ID4 in breast cancer and recruit lncRNA MALAT1 to regulate the splicing of VEGFA pre-mRNAs, consequently increasing the production of pro-angiogenic VEGFA isoforms [84]. Expression of mutp53 in bone marrow stromal cells increases VEGF synthesis by direct induction of its promoter and activation of protein kinase C (PKC) [53]. It has been reported in the literature that mutant p53 upregulates Prostaglandin Endoperoxide Synthase 2 (PTGS2), a key enzyme in prostaglandin E2 (PGE2) biosynthesis, which in turn promotes angiogenesis and immunosuppression [85].

(8) Mesenchymal stem cells-mediated mechanism

**Figure 1:**
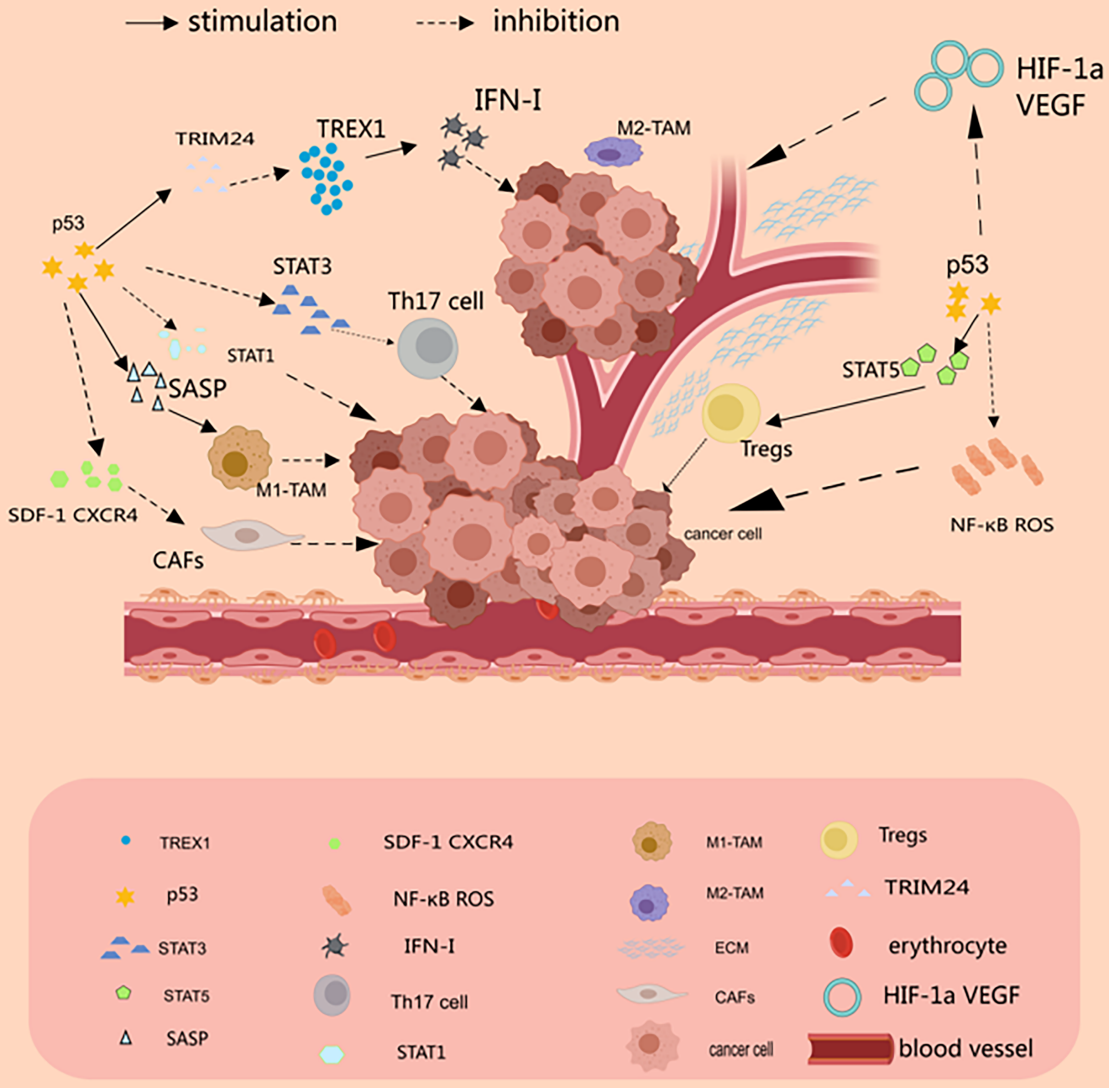
p53 and the tumor microenvironment. p53 can inhibit tumor development by suppressing the inflammatory response. In tumor-associated T cells, p53 inhibits the STAT3-Th17 axis but promotes the STAT5-Treg axis, which supports Tregs and thus suppresses the autoimmune response. In CAFs, p53 protein inhibits the expression and secretion of SDF-1 in stromal fibroblasts, which in turn inhibits the invasive and migratory effects of its cognate receptor, CXCR4, on tumor cells. In TAM, p53 drives M1 macrophage polarization by inducing senescence and SASP. p53 inhibits angiogenesis by binding to SP1 protein or degrading HIF-1α (Drawn by MedPeer).

There is growing evidence that MSCs are involved in the formation of TME and the promotion of tumor growth [86]. MSCs are a critical part of TME, which promotes tumor proliferation and invasive metastasis, angiogenesis, and even tumor drug resistance [86]. It has been reported in the literature that MSCs promote tumor progression by protecting tumor cells from senescence. Moreover, in colorectal cancer, MSCs regulate the p53/p21 pathway by decreasing the p53 half-life and thus functioning [87]. However, in the presence of inflammatory cytokines, p53-deficient MSCs exhibited enhanced immunosuppressive capacity [88]. Fig. 1 and Table 1 summarize the interactions between different states of p53 and the tumor microenvironment (Fig. 1 and Table 1).

**Table 1:** List of different p53 states affecting the tumor microenvironment

TME	Effects of different p53 status-mediated signaling pathways on the tumor microenvironment				References
	Wild-type p53-mediated pathway	Mut-type p53-mediated pathway	p53 inactivation-mediated pathway	p53 deletion-mediated pathway	
Chronic inflammation-related mechanism	p53 inhibits the NF-κB pathway thereby suppressing the expression of cytokines and chemokines and reducing the accumulation of ROS.	p53 mutant proteins can participate in the cGAS-STING-TBK1-IRF3 signaling pathway to inhibit tumor immunosurveillance, thereby eliminating IFN-I responses and innate immune activation.	p53 inactivation triggers overactivation of the NF-κB pathway, which induces increased secretion of inflammatory cytokines and chemokines, and ultimately exacerbates chronic inflammation and tumorigenesis.	p53 deletion increases ROS levels, activates STAT3 signaling.	[17,18,25,28]
	wtp53 activates the cGAS/STING pathway, induces IFN-Is, and produces tumor suppression.				
ECM-related mechanism		Ectopic expression of mutant p53 variants R175H, R273H and R280K in MCF10A cells stably silenced wt-p53 and increased miR-30d expression, promoting tumor growth and metastatic colonization.			[30]
Tumor-infiltrating T cells-mediated mechanism	p53-STAT3-Th17 axis and p53-STAT5-Treg axis support Tregs, thereby suppressing the autoimmune response.				[39,40,45]
	The p53-MDM2 pathway enhances STAT5 stability in tumor-infiltrating CD8^+^ T cells and controls T cell immunity.				
Mesenchymal stem cells-mediated mechanism	MSCs regulate the p53/p21 pathway by decreasing the p53 half-life and thus functioning.				[87]
Tumor-associated macrophages-mediated mechanism	p53/MDM2/c-Myc axis promotes M2 macrophage polarization.	Tumor cells carrying p53R273H or p53R249S mutants induced macrophages to enter the M2 state.	Inactivation of p53 leads to an overreaction of the STAT1 pathway, resulting in increased production of inflammatory cytokines.		[41,63,68]
TANs-mediated mechanism		The gain-of-function mutant Trp53R172H causes tumor-associated neutrophil infiltration.		The p53 gene deletion increases neutrophil levels through uncontrolled WNT signaling, leading to metastasis.	[75,80]
		p53R172H mutation may increase CXC chemokine levels through NF-κB.			
Angiogenesis-related mechanism		Mutp53 R175H and R273H bind ID4 in breast cancer and recruit lncRNA MALAT1 to regulate the splicing of VEGFA pre-mRNAs, consequently increasing the production of pro-angiogenic VEGFA isoforms.			[84]

## Clinical Treatment and Drug Research

With in-depth research on the role of TME in drug resistance and tumorigenesis, more and more studies are aimed at targeting TME to improve the therapeutic efficacy against cancer. Targeting TME has advantages over directly targeting cancer cells. This is due to the fact that cancer cells have an unstable genome and are prone to drug resistance, whereas the genes of non-oncogenic cells in TME are more stable and vulnerable [89–91]. p53 is associated with the induction of immunogenic cell death, antigen processing and cytokine production, immune checkpoint regulation, and immune tolerance [92]. Therefore, p53 and TME have gained widespread attention in research and clinical drug development as important factors in ecological and evolutionary processes during tumorigenesis and therapy (Table 2).

**Table 2:** List of drugs targeting p53 and TME

Target	Drug	Mechanism	Diseases	Stage of development	Clinical trial number	References
Tumor-associated T cells	Ipilimumab	Reactivation of the immune system	MSI-H deficient mismatch repair (dMMR) metastatic colorectal cancer (mCRC), TNBC, Melanoma	Clinical phase II, Clinical phase III		[52,93]
	Pembrolizumab	Blockage of PD-L1 and PD-1	mCRC			[52]
	Nivolumab	Blockage of PD-L1 and PD-1	mCRC			[52,94]
TAMs	PF-04136309	CCR2 inhibitor	Pancreatic ductal adencarcinoma	Phase Ib	NCT02732938	[95]
	RP-182	Reprogramming of M2-like TAM to an anti-tumor m1-like TAM phenotype				[96]
	MLN1202	Anti-ccr2 antibody	Bone metastasis	Clinical phase II	NCT01015560	[7]
p53	Curcumin	Induction of apoptosis				[17,97]
	SCH529074	Restoration of the growth inhibitory function of mutant p53				[52]
	ONYX-015	Induction of apoptosis				[89]
	CP31398	Restores wild-type conformation		Preclinical		[52]
	PRIMA-1	Restores wild-type conformation				[52]
	APR-246	Mutant p53 refolds to wild-type p53	Ovarian cancer medullary tumor esophageal cancer	Clinical phase II	NCT02098343	[52]
					NCT03072043	
					NCT02999893	
	Ad-p53	Exogenous p53				[98]
	MIRA-3	Restoration of p53 function				[89]
	PK7088	Elevation of p21 and NOXA levels		Preclinical		[52,99,100]
	ZMC-1	Restoration of normal folding and transcriptional activity in p53 mutants				[52,101]
	COTI-2	Restoration of folding and function of p53 mutants		Clinical phase I		[100]
	P53R3	Recovery of specific p53 mutants	Glioma cells			[102]
	Bispecific antibody (H2)	Specific targeting of TP53R175H		Preclinical		[52]
p53-MDM2	Chlorofusin	Disruption of p53-MDM2 interaction	Liver cancer			[98]
	APG115	Separation of MDM2 and p53				[45]
	HDM201	MDM2 inhibitor				[52,92,103]
	Nutlin-3a	MDM2 antagonist				[89,103]

(1) Tumor-associated T cells targeted drugs

Immune cells provide better ideas for the development of anticancer therapies based on targeted immune components [12]. CD8^+^ T cells are one of the determinants of treatment efficacy and prognosis in cancer patients [45,104]. Current treatments for TME focus on T cells, such as checkpoint blockade and chimeric antigen receptor (CAR) T cell therapy [3]. In addition, targeting p53-MDM2 interactions has also been shown to contribute to the enhancement of anti-tumor immunity in CD8^+^ T cells [12,45,105,106].

Immune checkpoint inhibitors (ICIs) produce longer-lasting anti-cancer effects by enhancing the anti-tumor response of T cells [38]. Studies have indicated that ICIs play a pivotal role in the treatment of cancers such as melanoma and renal cell carcinoma [93]. Among these, programmed cell death ligand 1 (PD-L1), a potential therapeutic target, has high mutational activity and is overexpressed in 20% of triple-negative breast cancer (TNBC) patients [107]. Mu et al. evaluated the intensity of PD-L1 expression in 109 non-small cell lung cancer (NSCLC) specimens. Immunohistochemical analysis revealed a strong correlation between PD-L1 expression and shorter survival time in adenocarcinoma patients [108]. Alteration of PD-L1 on tumor cells leads to suppression of the immune response by binding its receptor PD-1 on CTL [52]. Among PD-1-targeting antibodies (anti-PD1), both Pembrolizumab and Nivolumab bind to PD-1 and block interactions with PD-L1, with encouraging results in mCRC progressing on chemotherapy [52,94]. Mutated p53 has been reported to be associated with immune cell infiltration and reduced PD-L1 expression [109]. It has been found that p53 activation in combination with immune checkpoint blockade therapy is a breakthrough in the treatment of many tumors. *In vitro* analysis by Yoon et al. showed that p53 is involved in the expression of PD-L1 on cancer cells, thereby interacting with T cells and evading immune surveillance [110]. One study reported a complete clinical remission in a patient with metastatic TNBC treated with a combination of ipilimumab and nivolumab (PD-1 antibody), as well as IL-2 and local and systemic hyperthermia [111]. In addition, a study reported complete clinical remission in a patient with metastatic TNBC after receiving a combination of ibritumomab and nivolumab (a PD-1 antibody), IL-2, and local and systemic thermotherapy [112]. A study reports the distribution of a spatiotemporally controllable synergistic bacterial-directed therapy in tumor tissues to reverse the immunosuppressive microenvironment. The bacteria were first synchronously remodeled from the inside out to express photothermal melanin (Bac^Mel^). An immune checkpoint inhibitor (αPD-1) was then deposited on the surface of Bac^Mel^, which was pre-coated with polydopamine (B-αPD-1). The immunosuppressed tumor microenvironment was synergistically reprogrammed by the dual immune activation effects of photothermal stimulation and immune checkpoint inhibition. As expected, tumor growth was significantly inhibited under Bac^Mel^ + NIR irradiation in both subcutaneous and ectopic 4T1-tumor mouse models, and the survival time of mice was prolonged in both cases. Considering future translations, some potential challenges, such as mode of administration, frequency and dose of modified bacteria, mechanism of bacterial distribution and safety issues, should be systematically evaluated [113].

(2) Macrophages targeted drugs

Macrophages play a great role as targets for antitumor therapy. A key feature of macrophages is their plasticity; therefore, reprogramming TAM to an anti-tumor phenotype is a very promising strategy for tumor therapy. Anti-tumor macrophages (M1-like) have the ability to scavenge and destroy tumor cells. RP-182 selectively induces a conformational switch of the mannose receptor CD206 expressed on TAMs expressing the M2 phenotype, reprogramming M2-like TAMs to an anti-tumor m1-like TAM phenotype [96].

It has been found that Curcumin inhibits p53 mutation-induced macrophage induction and M2-promoted tumorigenesis [17]. In addition, studies have shown that curcumin can impede tumor progression by restoring T-cell populations, altering TAM characteristics, and reducing Treg [97].

The CCL2-CCR2 axis is a target for the development of drugs against TAM. CCL2 is known to be a potent chemoattractant for monocytes or macrophages. Results from a phase 1b study of the human CCR2 small molecule antagonist, PF-04136309, in combination with chemotherapy (NCT02732938) showed no benefit of chemotherapy in the treatment of metastatic PDAC [95]. In addition, an anti-CCR2 antibody (MLN1202) was shown to be effective in 14 of 43 patients with bone metastases in a phase II clinical trial (NCT01015560) in metastatic cancer [114].

CD47 is another potential target for enhancing the anti-tumor activity of macrophages. In preclinical studies and early clinical trials, blockade of the signal regulatory protein alpha (SIRPα)-CD47 axis by anti-CD47 antibodies or recombinant SIRPα crystal fragment (Fc) fusion proteins increased the tumor killing capacity of macrophages [96].

(3) p53 targeted drugs

As a nuclear transcription factor, p53 does not have the typical characteristics of a drug target and has therefore long been considered undruggable. Nevertheless, several promising p53-based therapies have emerged in recent years, and have been shown to be effective in preclinical models [115]. For tumors with missense mutations in TP53, drug development has focused on compounds that restore the wild-type conformation and activity of the mutp53 protein. In contrast, for cancers that maintain wtp53, the main approach has been to identify small molecules that liberate p53 from the inhibition of its negative regulators, such as MDM2, which in turn releases the full activity of p53 [115].

A study by Nemunaitis et al. of a clinical trial combining Ad-p53 (exogenous p53) with chemotherapy suggests that elevated p53 activity in TME may maintain p53-functioning stromal cells, thereby enhancing antitumor efficacy [116]. However, the clinical outcomes of Ad-p53 therapies have been unsatisfactory. To improve delivery efficiency and take advantage of the inactivating properties of p53 in many tumors, more advanced vector systems have been developed. For example, the oncolytic adenovirus ONYX-015 replicates and induces apoptosis only in p53-deficient tumors. However, to date, these viruses have had limited efficacy [89].

Cysteine binding compounds such as CP31398, APR-246 refold p53 mutants into wild-type conformation [52]. Among them, PRIMA-1 is a p53-independent negative regulator that enhances the thermal stability of mutant p53, thereby promoting the refolding of mutant p53 into the p53 wt conformation and enhancing the effect of immune checkpoint blockade [52]. In multiple phase II clinical trials, APR-246 was used in combination with carboplatin chemotherapy in patients with p53-mutated plasma ovarian cancer (NCT02098343), APR-246 in combination with azacitidine for p53-mutated medullary tumors (NCT03072043), and APR-246 in combination with 5-FU and cisplatin for esophageal cancer (NCT02999893) [52]. A recent phase II trial on APR-246 plus azacitidine as post-transplant management for the treatment of patients with MDS and AML with TP53 mutations found a 1-year relapse-free survival (RFS) of approximately 30% after transplantation and a median overall survival (OS) of 5–8 months after transplantation, and an RFS of 58% for the combination of APR-246 and azacitidine, as compared with previous trials. The median OS was 19.3 months [115]. In addition, based on crystal structure and computational analysis, MIRA-3 was developed, which effectively activates downstream apoptosis-inducing targets by transforming mutant p53 and restoring p53 function [89]. In addition to cysteine-binding compounds, small molecule drugs such as PK083 and PK7088 can specifically bind to the surface cavity of Y220C p53 mutants, inducing p53 refolding into a wild-type conformation [99].

Normal folding of wtp53 requires zinc as a cofactor. It was found that adding zinc to cells restored the ability of p53 mutants to bind zinc, thereby inhibiting tumor development [101]. Zinc metallochaperone-1 (ZMC-1), also named NSC319726, was found to restore normal folding and transcriptional activity in p53 mutants [52]. ZMC-1 was found to promote tumor cell apoptosis in xenograft model mice carrying the specific p53 mutation R172H [101]. In addition, another Zn2+ chelating compound, COTI-2, restored the folding and function of the p53 mutant. Although the exact mechanism is still under investigation, COTI-2 has been tested in phase I clinical trials in gynecologic and head and neck cancers. In addition, the small molecule drug P53R3 has been reported to restore the DNA-binding ability of specific p53 mutants, such as 175H, R273H, and M237 [100]. A study evaluated the efficacy of intraperitoneal injection of COTI-2 to inhibit the growth of HT-29 and SHP-77 xenografts in immunocompromised mice *in vivo*. COTI-2 significantly inhibited tumor growth of HT-29 human colorectal tumor xenografts at a dose of 10 mg/kg. In addition to reducing tumor volume at a specific time after treatment, COTI-2 delayed the time required for tumors to reach a specific volume. In the case of SHP-77 xenografts, it was not possible to calculate the COTI-2-induced time delay to reach a similar volume because these tumors never reached an average volume similar to that of the controls. In fact, COTI-2 was significantly more effective than cisplatin and paclitaxel in inhibiting the growth of SHP-77 xenografts [117].

In addition the TP53 mutant R175H was targeted with the highly specific antibody H2, which binds to the human leukocyte antigen-a (HLA-A) allele on the cell surface, while another of its structural domains binds to the T cell receptor. This bispecific antibody proved effective in preclinical models, inducing regression of early mouse and established human xenograft tumors [52].

Furthermore, MDM2 is a very attractive therapeutic target, especially in cancers with wild-type p53 [105,106]. MDM2 is not only an antagonist of p53, but it is also essential for modulating CD8^+^ T cell-mediated antitumor immunity [12,45]. Studies have shown that MDM2 can stabilize STAT5 levels by reducing c-Cbl-mediated STAT5 degradation. Therefore, targeting p53-MDM2 interactions can enhance MDM2 function in T cells and thereby improve CD8^+^ T cell-mediated anti-tumor immunity [12,45]. In fact, a number of inhibitors of p53-MDM2 interactions are in clinical development. The mechanism of Chlorofusin for the treatment of hepatocellular carcinoma is that it binds to MDM2 and targets the p53-MDM2 interaction, thereby ensuring stabilization of p53 activity [98]. In studies related to ovarian cancer, targeting of MDM2 by AMG 232 resulted in activation of p53 in MDM2-overexpressing ovarian cancer cells and sensitized them to T cell-mediated killing [98,118]. AMG 232 in combination with cytotoxic chemotherapy has shown higher anti-tumor efficacy compared to AMG 232 or chemotherapy alone. In recent years, more than 10 clinical trials have been initiated with AMG 232, including a phase III trial of myelofibrosis after failure of JNK inhibitors [115]. Another study showed that the APG115 drug resulted in the dissociation of MDM2 and p53, leading to MDM2 expansion in T cells [45]. It was found that HDM201, an MDM2 inhibitor, impeded p53 degradation and promoted the ratio of CD8^+^ T cells to Tregs, thereby improving the immune-involved antitumor response and thus controlling the crosstalk between tumors and immune cells [92]. Studies have shown that Nutlin-3a destabilizes the p53/MDM2 complex, and increases p53 activation [119]. Another study reported that SCH529074 inhibited p53 degradation by decreasing MDM2-mediated p53 ubiquitination and that SCH529074 bound the core structural domains of p53 mutants and increased the expression of p53 target genes [52]. All of the above reports demonstrate that targeting the mechanism of p53-MDM2 interaction is expected to be a therapeutic approach to induce tumor cell death. However, cancer therapeutic applications are challenged by gaps in substantive knowledge of the p53-MDM2 pathway in the fields of tumor biology and oncology.

## Conclusions

Tumor suppressor p53-mediated cell cycle arrest, apoptosis, and senescence influence cancer progression [8]. With the increasing research on p53, other anti-tumor pathways of p53 have been continuously explored [8]. A growing body of research recognizes that p53 activity may regulate host immune function and modulate the immune landscape of the TME [11]. For example, p53 has been shown to induce antitumor immune responses through transcriptional regulation of genes encoding key cytokines, chemokines, and pathogen recognition. p53 may also play a key role in inhibiting the polarization of tumorigenic M2 tumor-associated macrophages, thereby promoting antitumor immunity [120–122]. Thus, it can be said that p53 plays a tumor suppressor role to a large extent by regulating immunity. Unlike wild-type p53, different states of p53 interact differently with the TME. most mutations in TP53 are missense mutations, such as the p53 R175H and R273H mutations. In addition to loss of tumor suppressor function, these mutants usually have gain-of-function activity and contribute to the malignant properties of cancer cells [9]. Mutp53 promotes tumor neoangiogenesis. In NSCLC, mutp53 promotes the expression of the pro-angiogenic factors IL8 and GRO-α through activation of inhibitor of DNA binding 4 (ID4) and hence, GRO-α. However, deletion of mutp53 impairs ID4 expression [9]. The main cause of p53 inactivation is mutations in the p53 DBD, which prevents the protein from binding to target DNA [27]. Inactivation of p53 in immune cells enhances the production of chemokines and inflammatory cytokines, promotes the differentiation and function of Th17 cells, and inhibits the differentiation of Tregs [39,41]. The specific mechanism of p53-mediated immune surveillance of tumor cells is not yet fully understood, and the mechanism of interaction between p53 and TME needs to be investigated further. For example, more in-depth studies on the interaction mechanism between p53 and human TANs are lacking due to the limited extraction techniques of human TANs [74]. The mechanism of interaction between different states of p53 and the tumor microenvironment also needs to be further investigated.

**Figure 2:**
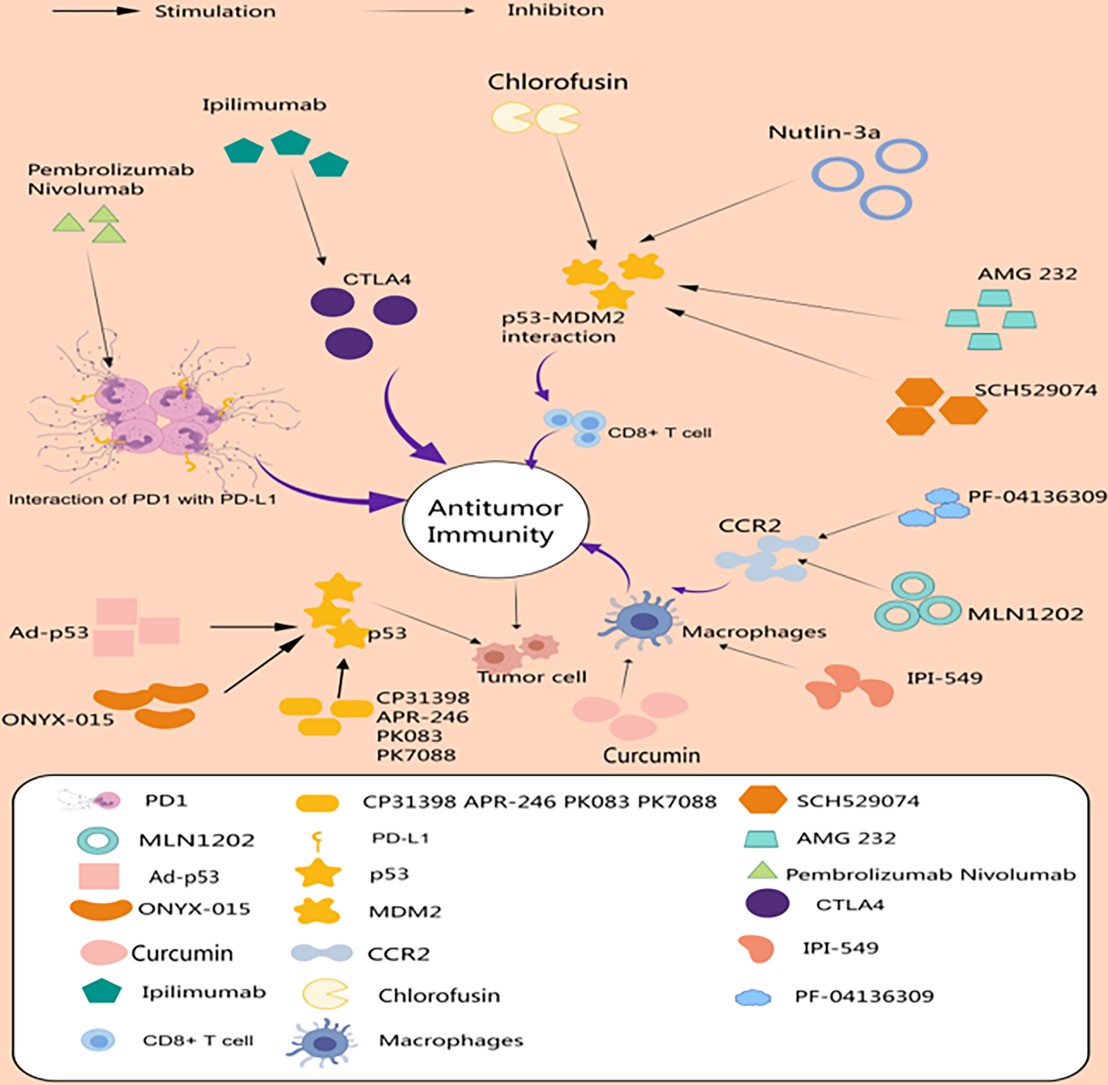
Drugs targeting p53 and the tumor microenvironment. The immune checkpoint blockers Pembrolizumab, Nivolumab, and Ipilimumab enhance anti-tumor immunity by boosting T-cell effects. AMG 232, SCH529074, Chlorofusin, and Nutlin-3a targeting p53-MDM2 act through CD8^+^ T cell-mediated antitumor immunity. Ad-p53, ONYX-015, and others exert tumor suppressor effects by enhancing p53. Curcumin, IPI-549, PF-04136309, and MLN1202 exert antitumor effects by targeting macrophages (Drawn by MedPeer).

p53 and the tumor microenvironment are important factors in evolutionary and ecological processes during tumorigenesis and therapy. A growing number of studies have attempted to target p53 and components of TME to enhance therapeutic efficacy against cancer (Fig. 2). Drugs targeting p53 achieve tumor suppression by activating p53, restoring the wild-type conformation of p53, and reducing ubiquitination of p53. Targeting TME has advantages over directly targeting cancer cells, which have an unstable genome and are prone to drug resistance, whereas the genes of non-oncogenic cells in TME are more stable and vulnerable. Above we have described drugs that target tumor-associated T cells, target macrophages, and target p53. Among them, in clinical treatment, drugs targeting p53 include those activating p53, such as Ad-p53 and ONYX-015; those restoring the wild-type conformation of p53, such as APR-246 and PRIMA-1; and those targeting p53-Mdm2, such as AMG 232 and HDM 201. Among them, drugs targeting p53-Mdm2 not only stabilize the level of p53, but also play an important role in regulating CD8^+^ T cells [52]. The lack of in-depth knowledge of the tumor microenvironment has hindered the discovery and development of drugs targeting the tumor microenvironment. In terms of clinical treatment, immunotherapies have produced meaningful responses in many cancers. Factors identified as contributing to clinical failure of immunotherapy include tumor heterogeneity, low tumor mutation burden, immunosuppressive tumor microenvironment, and associated low tumor cytotoxicity T-cell infiltration [123]. Therefore, TME-targeted therapy may need to be combined with other treatments to maximize efficacy and benefit more patients.

## Data Availability

Not applicable.
